# Educating Asthmatic Children in European Ambulatory Pediatrics: Facts and Insights

**DOI:** 10.1371/journal.pone.0129198

**Published:** 2015-06-10

**Authors:** Marie Noëlle Robberecht, Laurent Beghin, Antoine Deschildre, Valérie Hue, Laura Reali, Vesna Plevnik-Vodušek, Marilena Moretto, Sigurlaug Agustsson, Emile Tockert, Elke Jäger-Roman, Dominique Deplanque, Abolfazl Najaf-Zadeh, Alain Martinot

**Affiliations:** 1 European Confederation of Primary Care Pediatricians (ECPCP), 11 quai Général Sarrail, 69006 Lyon, France; 2 Association Française de Pédiatrie Ambulatoire, pediatric office, 32 avenue Desrousseaux, 59370 Mons-en-Baroeul, France; 3 Centre d’Investigation Clinique -9301-Inserm, CHRU, F-59037 Lille, France; 4 Inserm U995, IFR 114, Univ Nord de France, F-59037 Lille, France; 5 Clinique de Pédiatrie, Hopital Jeanne de Flandre, Av Eugène Avinée, F-59037 Lille, France; 6 Associazione Culturale Pediatri, via Montiferru, 6–09070 Narbolia (OR), Italy; 7 Delovna slupina za primarno pediatrijo, Zdravstveni dom velenje Vodnikova 1, 3320 Velenje, Slovenia; 8 Pediatric Department Hôpital St Pierre- Université Libre de Bruxelles (ULB)- Rue Haute 322 à 1000, Bruxelles, Belgique; 9 Société Luxembourgeoise de Pédiatrie, Center of Pediatrics, Val Sainte Croix, Luxembourg; 10 Berufsverband der Kinder-und Jugendärzte, Köhlerstr. 23, Berlin, Germany; 11 EA 2694, Université Droit et Santé Lille (UDSL), Univ Lille Nord de France, Lille, France; St Francis Hospital, UNITED STATES

## Abstract

The aim of this study was to assess the role of European ambulatory pediatricians in caring for asthmatic children, especially in terms of their therapeutic education. We developed a survey that was observational, declarative, retrospective and anonymous in nature. 436 ambulatory pediatricians in Belgium, France, Germany, Italy, Luxembourg and Slovenia were asked to participate in the survey providing information on three children over 6 years old suffering from persistent asthma, who had been followed for at least 6 months. We considered the pediatricians’ profile, and their role in the therapeutic education of children. 277 pediatricians (64%) responded: 81% were primary care pediatricians; 46% participated in networks; 4% had specific training in Therapeutic Patient Education; 69% followed more than 5 asthmatic children per month, and over long periods (7 ± 4 years). The profiles of 684 children were assessed. Answers diverged concerning the provision of a Personalized Action Plan (60–88%), training the child to measure and interpret his Peak Expiratory Flow (31–99%), and the prescription of pulmonary function tests during the follow-up programme of consultations (62–97%). Answers converged on pediatricians’ perception of their role in teaching children about their condition and its treatment (99%), about inhalation techniques (96%), and in improving the children’s ability to take preventive measures when faced with risk situations (97%). This study highlights the role of European pediatricians in caring for asthmatic children, and their lack of training in Therapeutic Patient Education. Programmes and tools are required in order to train ambulatory pediatricians in Therapeutic Patient Education, and such resources should be integrated into primary health care, and harmonized at the European level.

## Introduction

Asthma is the most common chronic illness in children in the European Union, with 10–12% of children affected [1]. Asthma diminishes the quality of life of the child and his family [2]. In 2007, the standardized annual hospitalisation rate for asthma in France was 8 per 10,000 [3]. In 2004, the overall cost of the condition in Europe was more than €3bn [4].

For several years now, pediatricians have had guidelines proposed by academic societies for dealing with asthma [5–10], including the therapeutic education of children. The concept of Therapeutic Patient Education (TPE) was defined in a World Health Organization report published in 1998: the aim of this pedagogical initiative is “to help patients and their families to acquire or maintain the competencies that they need to live with a chronic illness” [11].

Numerous studies have established the link between the TPE of the asthmatic child and his family, and a significant improvement in the level of control of the disease and the resulting in terms of quality of life [12–15]. The effect was more pronounced in programmes aimed at individual guidance.

The practices of the European ambulatory pediatricians in the field of the education of asthmatic children and their families have not yet been evaluated, much less harmonized. The European Confederation of Primary Care Pediatricians (ECPCP), which represents 70% of all ambulatory pediatricians in 19 European countries, has therefore conducted a survey. Its primary objective was to evaluate the pedagogical approach of European ambulatory pediatricians when caring for asthmatic patients, and the means available to do so (training, qualification, equipment in the medical office), based on official guidelines [5–10]. The secondary objective was to examine whether ambulatory pediatrician practices differ according to country and/or whether the pediatrician is a member of a network.

## Materials and Methods

This European observational, retrospective, declarative and anonymous study was initiated and coordinated on a voluntary basis by members of the research working group of the ECPCP. Countries participated in the study when one of those members took on the role of national referent. Participating countries were: Belgium, France, Germany, Italy, Luxembourg and Slovenia. Each national referent drew up a list of 60 to 100 eligible pediatricians consecutively taken from the membership list of his national society affiliated to the ECPCP. Luxembourg was an exception: all pediatricians were asked to cooperate, given their small number. In France, Slovenia, Italy and Germany, preference was given to one region. In Belgium, the sample included pediatricians from the French-speaking as well as from the Dutch-speaking community.

Each pediatrician was asked to include children on the basis of patient files from the last three months, concerning the three most recent children suffering from asthma requiring long-term treatment by inhaled anti-inflammatory medication. The children had to be at least six years old, and needed to have been seen by the pediatrician for at least six months in relation to their asthma.

The study comprised two questionnaires consisting of closed questions requiring simple answers. One questionnaire, the pediatrician’s record, concerned the data necessary to specify his/her action in the TPE: number of years of practice, qualifications (diploma certifying competence in TPE, pulmonology and/or allergology), membership of a network dedicated to allergology, and the devices and tools they had at their disposal, especially demonstration materials.

The second questionnaire concerned child characteristics, and the way in which the pediatrician dealt with the TPE. There were questions about knowledge, skills and behaviour. *Knowledge*: what does the child know about long-term treatment versus treatment of the attacks, early warning signs, signs of severity, and the “Personalised Action Plan” (PAP) (document explaining how to deal with the condition, including the course of action to be taken in the event of an attack, and when to call for the doctor or the emergency services). *Skills*: the child’s ability to use inhaling devices, a Peak Flow meter, and what measures to adopt depending on the Peak Expiratory Flow (PEF). *Behaviour*: the child’s ability to take specific and non-specific preventive measures.

The questionnaires were drawn up in French, and then translated into the languages of each country, with a triple validation procedure: translation from French into the required target language, verification of the technical terminology by a native pediatrician, translation from the target language back into French. The “Centre d’Investigation Clinique” in Lille numbered the questionnaires with a specific code for each country and sent them by post to the National referents. Each of them made the list of eligible pediatricians anonymous by transforming the names into numbers, sent the questionnaires to the pediatricians and checked their reception. All answers were sent back by post in numbered envelopes to the Centre d’Investigation Clinique. This procedure ensured the anonymity of participants, with neither the analysors nor the authors able to identify individuals. The data gathering took place simultaneously in the six countries between 5 January and 15 June 2009, with three reminders.

This non-interventional study was approved with waiver of written informed consent by the institutional review board of the University Hospital of Lille. According to French medical research regulation, this study did not require any declaration to a competent authority of personal information technology data. Pediatricians participating in this study provided verbal consent to the research working group of the ECPCP, verbal consent was centralised at the level of each country ECPCP representative. Children’s parents/guardians provided verbal consent to the ambulatory pediatricians, which was documented in the patient medical office record.

The study was descriptive and analytical with regard to country, membership of a network dedicated to allergology, and additional specialty in pulmonology and/or allergology. Qualitative variables were compared using the χ^2^ test, quantitative variables by using the ANOVA test. A probability level of p<0.05 was considered as significant. Odds ratios (OR), percentages and 95% confidence intervals (CI) were computed using Epi-Info 6.04fr (Centers for Diseases Control and Prevention, Atlanta, Ga, USA), and figures were rounded up to the nearest whole number.

## Results

Among the 436 pediatricians who received the questionnaires, 277 responded (64% response rate; ranging from 32% in Germany to 95% in Italy), of which 81% were primary care pediatricians and 46% were active within a network. Characteristics of the respondents are shown in Table 1. Four percent of responding pediatricians (from 0% in Belgium, Italy, and Luxemburg to 33% in Germany) were trained in TPE. Forty percent of pediatricians followed children in collaboration with allergologists (from 10% in Germany to 60% in Slovenia), 24% with pulmonologists (from 13% in Belgium to 37% in Luxembourg), 8% with the general practitioner (from 1% in Italy to 28% in Belgium), and 32% alone (from 24% in Slovenia to 51% in Germany). Table 2 summarizes the office equipment of the responding pediatricians. Altogether, 684 children were included; their characteristics and follow-up visits are shown in Table 3. Ninety-nine percent of respondents declared that they made sure that the child and his family knew the early warning signs and the indications of the seriousness of an attack, and 80% provided a written PAP after having explained it. Networking pediatricians provided a PAP more often than non-networking pediatricians (83% vs 76%, OR = 1.6; 95% CI:1.1–2.5). If a peak-flow meter had to be prescribed, 67% of respondents made certain that the child was able to use it, or demonstrated its use. Sixty percent made sure that the child knew his optimum PEF and what measures to take depending on the PEF measured. Networking pediatricians more often explained how to measure the PEF than non-networking pediatricians (78% vs 42%, OR = 4.4; 95% CI: 3.0–6.5).

**Table 1:** Characteristics of study pediatricians per country.

Country	Pediatricians contacted, n	Pediatricians responding, n (%)	Years in practice, mean (SD)	Subspecialist in Pulmo/ allergology (%)	Active within a network (%)	Seeing > 5 asthmatic children / month (%)	Trained in TPE (%)
Germany	75	24 (32)	18 ± 9	33	79	96	33
Belgium	60	37 (62)	19 ± 9	27	54	80	0
France	80	52 (65)	25 ± 7	15	41	44	2
Italy	100	95 (95)	23 ± 4	6	20	35	0
Luxemburg	46	16 (35)	13 ± 8	12	0	69	0
Slovenia	75	53 (71)	15 ± 8	38	90	84	6
**Total**	**436**	**277 (46)** ^a^	**20 ± 8** ^b^	**19** ^c^	**46** ^a^	**60** ^d^	**4** ^a^

SD = standard deviation; TPE = Therapeutic Patient Education;

^a^ p < 10^–8^,

^b^ p < 10^–6^,

^c^ p<10^–4^,

^d^ p < 10^–5^

**Table 2:** Office equipment of the responding pediatricians per country.

Country	Prick tests, %	Peak Flow Meter, %	Spirometer, %	Demonstration material for inhaling devices, %
Germany	79	100	87	100
Belgium	76	65	70	92
France	27	88	21	85
Italy	36	59	35	90
Luxemburg	31	69	25	87
Slovenia	4	100	23	92
**Total**	**37** ^a^	**78** ^a^	**39** ^a^	**90** ^ns^

^ns^ = not significant;

^a^ p < 10^–8^

**Table 3:** Characteristics of study patients and their follow-up visits per country.

				Assessment and monitoring				Patient education		
Country	Children, n	Age, y, mean± SD	Follow-up duration, y, mean± SD	Asthma control level (%)	Long-term treatment adaptation (%)	Treatment side-effects (%)	PFT every 12–18 months (%)	PAP (%)	PEF measuring (%)	Optimum PEF (%)
Germany	68	11 ± 3	8 ± 4	100	100	100	97	84	98	97
Belgium	94	10 ± 3	6 ± 4	88	94	93	75	81	49	35
France	106	10 ± 3	7 ± 4	91	93	92	62	60	72	62
Italy	232	10 ± 2	8 ± 3	87	87	91	77	83	37	31
Luxemburg	38	9 ± 3	6 ± 3	81	87	87	71	71	56	48
Slovenia	146	11 ± 3	6 ± 3	95	98	94	94	88	99	99
**Total**	**684**	**10 ± 3** ^a^	**7 ± 4** ^a^	**90** ^b^	**93** ^c^	**93** ^**ns**^	**80** ^d^	**80** ^a^	**67** ^d^	**60** ^d^

SD = standard deviation; PFT = pulmonary function test; PAP = personalised action plan; PEF = peak expiratory flow;

^ns^ = not significant;

^a^ p < 0.01,

^b^ p < 10^–2^,

^c^ p < 10^–3^,

^d^ p < 10^-^

The vast majority of respondents explained or made sure that the child and his family knew how to inhale the drugs (96%), and knew the difference between long-term treatment and treatment of the attack itself (99%), the situations that might trigger an asthma attack (97%), and the required preventive measures (98%). Pediatricians invited the child and his family to regular consultations for assessment and monitoring of asthma control (Table 3). Networking pediatricians were more likely than non-networking pediatricians to assess the level of asthma control (95% vs 86%, OR = 3.2; 95% CI: 1.7–6.2), adapt the long-term treatment (97% vs 88%, OR = 4.4; 95% CI: 2.0–9.7), and make certain that the pulmonary function test took place on a regular basis (89% vs 70%, OR = 3.3; 95% CI: 2.1–5.1).

## Discussion

This observational study is the first to highlight and compare European ambulatory pediatricians’ self-declared participation in the TPE of asthmatic children and their families. While responding pediatricians almost unanimously declared having taught the child to increase his knowledge of the condition, his skills and his capacity to adapt his behaviour in order to avoid a crisis, there were significant differences in the pediatrician pedagogical attitude, and in the content of the regular follow-up programme. Very few responding pediatricians were trained in TPE, although they cared for a large number of asthmatic children, on a long-term basis.

This study highlights the fact that ambulatory pediatricians have the necessary knowledge and equipment for child’s asthma management in the ambulatory setting. The vast majority of respondents scheduled regular asthma visits, and the mean follow-up of the asthmatic children was 7 years. Two previous studies with different objectives and methodology showed that the treatment, control and follow-up asthma among European children were insufficient [16–17]. Both studies were performed at a time when TPE had not yet been recognized as an essential part of the daily care of children suffering from asthma, and were based on data gathered from general practitioners or children and their families without specifying whether or not the patient was followed by a pediatrician. This could explain the contrasting results of these studies.

However, in our study, significant differences regarding pedagogical attitude of the respondents and their office equipment were notable. These are likely related to the differences in the characteristics of respondents and their practice setting. The heterogeneity for prick test and spirometer availability between countries probably reflects the difference in relative numbers between PCPs and pediatricians with subspecialty. We found also a significant difference in the figure concerning “Provision of written PAP” which is a basic indicator of efficient asthma management [18]. The high overall percentage of pediatricians issuing a PAP is encouraging, particularly in Slovenia and Germany where a “Chronic Disease Management Programme” had been launched. The greatest difference between countries concerned “The teaching of PEF measurement and its interpretation”. This could be explained by the controversial place of the PEF in asthma management, which is considered rather complex, although the GINA guidelines do state that “PEF measurements can be an important aid in both diagnosis and monitoring of asthma” [19].

TPE is an essential component for the efficient self-management and quality of care of all long-term diseases and conditions. It has been shown to be of beneficial effects in pediatric asthma [12–15]. In our study, only 4% of pediatricians were trained in TPE. Such training is a precondition for a pedagogical approach that is the only viable way that should lead to changes in patients’ behaviour. Those changes are necessary to acquire competencies of self-care and competencies to adapt to and control the illness [20]. However, as TPE implies a complete overhaul of the relationship between the carer and the patient, the pediatrician needs to be aware of what TPE is and how to implement it. In our study, the very low number of pediatricians trained in TPE could be related to the characteristics of respondents and their practice setting. In Europe, training programmes for TPE are proposed in the so-called asthma schools, and are basically aimed at specialised professionals or those working in a hospital environment, whereas, in our study, the vast majority of respondents were PCPs. Furthermore, it was in those countries with the highest rates of TPE training, Germany and Slovenia, that respondents were the most hyper-specialised, and that TPE was financially supported and integrated into protocols recommended by national health authorities for the care of asthmatic children [6,9].

TPE is an integral part of asthma care. Therefore, PCPs caring for children with asthma should be trained in TPE. Such training would be all the more efficient as PCPs care for a large number of asthmatic children, on a long-term basis, starting at an early age [21]. The long-term relationship of trust with the patient suggests that families will be more likely to adhere to the educational programmes proposed by their PCPs. Indeed, knowing his patient well, the PCP can easily identify the levers which facilitate the child’s learning and the inappropriate behaviours which could hinder the effective management of the child’s condition. This is all the more valid as the vast majority of the families declare they are willing to ensure the daily assistance of their members, in the field of prevention, care and first aid. A French study conducted amongst the general population in 2010 showed that more than 70% of the interviewed families acknowledged the need to be trained in the field of “Education to Care and Aid” [22]. The importance of collaborative goal setting and better partnering with the parents to achieving effective self-management has been acknowledged [23]. Indeed, the most important part of asthma education programmes is a high level of agreement between carer and patient regarding the goals of the management of the condition [24]. Consequently, the carer must learn how to develop structured programmes specifically adapted to each patient.

The implementation of TPE has been shown to be hampered by many practical problems: the heterogeneity of practices, the various degrees of involvement of professionals and patients, the unregulated modes of funding and organisation [25]. Consequently, health authorities insist on the importance of coordinated action to put in place ways of providing TPE at various levels, and also on the promotion of quality [25].

Our study has some limitations that need to be considered. First, there is no evidence that the sample of pediatricians is representative. Equally, however, there is no reason to suppose that sampling on a regional basis, as described in the material and methods section, led to a selection bias. Moreover, the method used to recruit doctors improved the feasibility of the study. Second, our results were quite homogeneous and positive when they concerned teaching the child how to improve his knowledge, skills and health behaviour. Nevertheless, the methodology used to reach these pedagogical objectives was not specified by the respondents, and the retrospective filling in of files may have led to a memory bias. The pediatrician may thus have overestimated their role. Finally, it is likely that the most accurate results were from countries with the greatest response rate, while the very low response rates in certain countries could lead to an overestimation of results.

In summary this study has highlighted the lack of training in TPE among European PCPs, training that is necessary to foster a genuinely educational attitude that transcends the purely informative. It is important that TPE programmes are organised in the surgery of the PCP, i.e. in the patients’ daily environment, integrated into primary health care, and linked with other health care professionals. These measures would make access to TPE easier for the families that most need it, and improve their compliance to long-term asthma management. It is therefore necessary to set up a rigorous and structured approach throughout Europe, specifically geared to the training of pediatricians, in their ambulatory practice, in TPE. It is in this regard that the ECPCP has launched a new project in ambulatory pediatrics, viz. the TEACHER project (Training to Educate Asthmatic CHildren in EuRope).
